# Deletion of caveolin‐1 attenuates LPS/GalN‐induced acute liver injury in mice

**DOI:** 10.1111/jcmm.13831

**Published:** 2018-08-22

**Authors:** Tsung‐Huang Tsai, Kabik Tam, Shu‐Fen Chen, Jun‐Yang Liou, Yi‐Chen Tsai, Yen‐Ming Lee, Tai‐Yu Huang, Song‐Kun Shyue

**Affiliations:** ^1^ Institute of Biomedical Sciences Academia Sinica Taipei Taiwan; ^2^ Institute of Cellular and System Medicine National Health Research Institutes Zhunan Taiwan; ^3^ Graduate Institute of Life Science National Defense Medical Center Taipei Taiwan

**Keywords:** acute liver injury, caveolin‐1, D‐galactosamine, lipopolysaccharide, toll‐like receptor 4

## Abstract

Acute hepatic injury caused by inflammatory liver disease is associated with high mortality. This study examined the role of caveolin‐1 (Cav‐1) in lipopolysaccharide (LPS) and D‐galactosamine (GalN)‐induced fulminant hepatic injury in wild type and Cav‐1‐null (Cav‐1^−/−^) mice. Hepatic Cav‐1 expression was induced post‐LPS/GalN treatment in wild‐type mice. LPS/GalN‐treated Cav‐1^−/−^ mice showed reduced lethality and markedly attenuated liver damage, neutrophil infiltration and hepatocyte apoptosis as compared to wild‐type mice. Cav‐1 deletion significantly reduced LPS/GalN‐induced caspase‐3, caspase‐8 and caspase‐9 activation and pro‐inflammatory cytokine and chemokine expression. Additionally, Cav‐1^−/−^ mice showed suppressed expression of Toll‐like receptor 4 (TLR4) and CD14 in Kupffer cells and reduced expression of vascular cell adhesion molecule 1 and intercellular adhesion molecule 1 in liver cells. Cav‐1 deletion impeded LPS/GalN‐induced inducible nitric oxide synthase expression and nitric oxide production and hindered nuclear factor‐κB (NF‐κB) activation. Taken together, Cav‐1 regulated the expression of mediators that govern LPS‐induced inflammatory signalling in mouse liver. Thus, deletion of Cav‐1 suppressed the inflammatory response mediated by the LPS‐CD14‐TLR4‐NF‐κb pathway and alleviated acute liver injury in mice.

## 1 INTRODUCTION

Acute liver failure is a complex and life‐threatening syndrome resulting from severe acute liver injury, which causes sudden and massive liver cell death and liver dysfunction, thereby leading to multiple organ failure.^1^, ^2^, ^3^ The sepsis‐induced inflammatory response mediated by dysregulated innate immunity is associated with direct hepatocyte injury by inducing cellular apoptosis.^4^, ^5^ Lipopolysaccharide (LPS) from Gram‐negative bacteria stimulates the innate immune response and is implicated in the pathogenesis of liver injury in patients with severe bacterial infection.^6^, ^7^ LPS interacts with CD14 and Toll‐like receptor 4 (TLR4) to activate inflammatory signals and induce secretion of pro‐inflammatory cytokines, which stimulates the infiltration of inflammatory cells into the liver.^4^, ^5^, ^8^ Cytokines such as tumour necrosis factor‐α (TNF‐α), interferon‐γ (IFN‐γ), interleukin‐1β (IL‐1β) and IL‐10 released from these inflammatory cells and from hepatic Kupffer cells are involved in LPS‐induced liver injury.^9^ In addition, LPS induced the expression of adhesion molecules such as intercellular adhesion molecule‐1 (ICAM‐1) and vascular cell adhesion molecule‐1 (VCAM‐1) in liver cells, which facilitated immune cell infiltration and exacerbated liver damage.^10^, ^11^

Caveolin‐1 (Cav‐1) is a cholesterol‐binding integral membrane protein and the major protein of caveolae, which function as transport carriers and signaling platforms on the plasma membrane. Cav‐1 is synthesized in endoplasmic reticulum and interacts with proteins to regulate protein activity, transportation and degradation.^12^, ^13^, ^14^ Cav‐1 regulates various functions and signalling pathways in liver, including lipid and glucose metabolism, mitochondria biology and hepatocyte proliferation.^13^, ^15^ A number of studies have shown an association of Cav‐1 with the inflammatory response. Deletion of Cav‐1 suppressed nuclear factor κB (NF‐κB) activation and lung injury in mice challenged with LPS.^16^ Cav‐1‐deleted mice showed attenuated serum secretion of cytokines, chemokines, TNF‐α, IFN‐γ and nitric oxide (NO) in mice infected with *Trypanosoma cruzi*.^17^ Moreover, Cav‐1 plays a key role in the monocyte‐to‐macrophage differentiation, and its ablation hindered CD14‐TLR4‐NF‐κB signaling and Gram‐negative bacterial phagocytosis in macrophages and mice.^14^, ^18^ However, other data suggest that deletion of Cav‐1 enhances the inflammatory response. Deletion of Cav‐1 increased mortality and the production of inflammatory cytokines, chemokines and NO in mice infected with *Salmonella enterica* serovar typhimurium.^19^ Moreover, deletion of Cav‐1 increased inducible NO synthase (iNOS) expression, NO and peroxynitrite production and liver damage in a mouse model of binge drinking‐induced liver damage.^20^ Hence, Cav‐1 may have different roles in the inflammatory response depending on the stimulus.

Despite a number of reports describing the regulatory role of Cav‐1 under various physiological and pathological conditions, whether Cav‐1 participates in LPS‐induced hepatic injury during acute inflammation and hepatitis remains to be explored. We examined the role of Cav‐1 in LPS/D‐galactosamine (GalN)‐induced acute liver injury in wild‐type (WT) and Cav‐1‐null (Cav‐1^−/−^) mice. GalN, an amino sugar, was used to inhibit cellular transcription activity to potentiate LPS toxicity, which causes liver cell apoptosis and necrosis and then hepatitis and liver dysfunction.^21^, ^22^ Our findings suggest that deletion of Cav‐1 suppresses LPS/GalN‐induced lethality, the inflammatory response and hepatic injury. The protective effect may be associated with decreased expression of TLR4, CD14 and adhesion molecules in liver of Cav‐1^−/−^ mice.

## 2 MATERIALS AND METHODS

### 2.1 Animals

All animal experiments were approved by the Institutional Animal Care and Utilization Committee of Academia Sinica. Eight‐week‐old male C57BL/6 mice (The National Laboratory Animal Center, Taiwan) and Cav‐1^−/−^ mice (Cav1^tm1Mls/J^) (The Jackson Laboratory, Bar Harbor, ME, USA) were housed and bred under specific pathogen‐free conditions. Cav‐1^−/−^ mice were backcrossed to C57BL/6 genetic background for at least 10 generations. Genotypes of Cav‐1^−/−^ mice were confirmed by PCR.

### 2.2 Reagents

LPS (*Escherichia coli 0111:B4*) and GalN were purchased from Sigma‐Aldrich (St. Louis, MO) and reconstituted in phosphate‐buffered saline (PBS). Enzyme‐linked immunosorbent assay (ELISA) kits for detecting TNF‐α, INF‐γ, IL‐1β, IL‐6, monocyte chemoattractant protein‐1 (MCP‐1) and macrophage inflammatory protein‐2 (MIP‐2) were from R&D Systems (Minneapolis, MN, USA). Antibodies (Abs) for cleaved caspase‐3 and caspase‐8 were from Cell Signaling (Beverly, MA, USA). The Ab anti‐F4/80 conjugated with fluorescein isothiocyanate (FITC) was from BioLegend (San Diego, CA, USA) and the Abs phycoerythrin (PE)‐labelled anti‐TLR4 and anti‐CD14 were from eBioiscience (San Diego, CA, USA). Abs for inducible nitric oxide synthase (iNOS) and Cav‐1 were from Santa Cruz Biotechnology (Santa Cruz, CA, USA). The Ab for F4/80 was from Invitrogen (Carlsbad, CA), Gr‐1 was from BD Pharmingen (San Diego, CA, USA) and VCAM‐1 was from Abcam (ab134047).

### 2.3 Murine model of acute hepatitis

LPS/GalN‐induced fulminant hepatitis in animals was created as previously described.^21^, ^23^, ^24^, ^25^ In brief, hepatic damage was induced by intraperitoneal injection of LPS (40 μg/kg) and GalN (400 mg/kg) in 200 μL. Saline was used for sham treatment. Mice were anaesthetized before surgery and were euthanized by suffocation with CO_2_. For survival test, mice were injected with LPS/GalN and monitored every 2 hours for 24 hours.

### 2.4 Analysis of liver alanine aminotransferase (ALT) and aspartate aminotransferase (AST) levels

Hepatocyte damage was examined by measuring plasma activities of AST and ALT with use of an automatic analyzer (Fuji Dri‐Chem Clinical Chemistry Analyzer, Japan).

### 2.5 Isolation of murine primary hepatocytes and liver Kupffer cells

Primary hepatocytes and liver Kupffer cells were isolated from mice by the in situ collagenase (type VI; Sigma‐Aldrich) perfusion technique, modified from that previously described.^26^, ^27^ In brief, liver was perfused with 37°C Ca^2+^ and Mg^2+^ free‐HBSS (Gibco, 14175‐095) containing 0.1 mmol/L EGTA for 10 minutes followed by liver digest medium (HBSS with 15 mmol/L HEPES, 5 mmol/L CaCl_2_, 0.13 mg/mL collagenase IV [Sigma‐Aldrich], 100 U/mL penicillin, and 100 μg/mL streptomycin) for an additional 10 minutes. The liver was carefully removed from the abdominal cavity. Liver fragments were shaken and teased gently in a Petri dish on ice in HBSS to free loose cells. The cell suspensions were filtered through a sterile 100‐μm nylon cell strainer (BD Biosciences) to remove undigested and connective tissue. Cells were centrifuged for 2 minutes at 50 × *g*, 4°C. The supernatant containing Kupffer cells was transferred to a new tube, and pellets containing hepatocytes were gently suspended in HBSS.

For Kupffer cell isolation, the supernatant was centrifuged at 70 × *g* for 3 minutes, and the pellet was discarded. The supernatant was centrifuged for 7 minutes at 650 × *g*; the pellet containing Kupffer cells was resuspended in 10 mL HBSS followed by two‐step Percoll gradient (25%/50%) centrifugation for 15 minutes at 1800 × *g*, 4°C. Cells from 25% to 50% Percoll gradient were collected and washed twice and cultured in RPMI‐1640 supplemented with 10% foetal bovine serum (FBS) and 2 mmol/L L‐glutamine for 2 hours. Non‐adherent cells were removed by replacing the culture medium. The purity of Kupffer cell cultures exceeded 98% by light microscopy, and viability was typically over 95% by trypan blue exclusion assay.

For hepatocyte isolation, 25 mL of pellet suspension was overlaid on 20 mL 90% Percoll solution in HBSS and mixed by gentle inversion and centrifuged for 5 minutes at 80 × *g*, 4°C. The pellet was washed twice, and purified hepatocytes were cultured in William's E medium supplemented with 10% FBS, 100 U/mL penicillin and 100 μg/mL streptomycin on Falcon cell culture dishes. The viability of hepatocytes was typically greater than 95% by trypan blue exclusion assay.

### 2.6 Quantitative real‐time PCR and RT‐PCR

Total RNA from whole liver was isolated by use of TRI reagent and underwent cDNA synthesis with oligo‐dT and SuperScript III reverse transcriptase (Invitrogen). The cDNA was used for RT‐PCR and quantitative real‐time PCR amplification with the TaqMan probe‐based real‐time quantification system (Applied Biosystems, Foster, CA, USA) with the primers listed in Table S1.

### 2.7 Histopathology and immunohistochemistry

Excised liver tissue was fixed in 10% neutral buffered formalin and embedded in paraffin. Sections (5 μm thick) were affixed to slides, deparaffinized and rehydrated, and stained with haematoxylin and eosin to determine morphology. Dehydrated slides were blocked with avidin and biotin and then incubated with rabbit Abs for cleaved caspase‐3 or caspase‐8, Gr‐1, or F4/80 at 4°C overnight. After repeated washing, slides were incubated with biotinylated goat anti‐rabbit Ab at room temperature for 30 minutes, washed and incubated with avidin‐biotin complex/horseradish peroxidase at room temperature for 30 minutes, then developed with diaminobenzidine as a substrate according to the manufacturer's instructions (DAKO, Carpintería, CA, USA).

### 2.8 Quantification of cytokine and chemokine levels

Plasma was harvested from WT and Cav‐1^−/−^ mice and stored at −80°C. The levels of TNF‐α, INF‐γ, IL‐1β, IL‐6, MCP‐1 and MIP‐2 were measured by ELISA kits.

### 2.9 Western blot analysis

A total of 30 μg cell lysates or nuclear extracts was resolved on 8%‐12% SDS‐PAGE and examined by Western blot analysis as described.^12^ β‐Actin was an internal control. All immunoblotting analyses were repeated at least three times with similar results. The protein levels were quantified by densitometry (Gel Pro v.3.1, Media Cybernetics).

### 2.10 Myeloperoxidase activity assay

Myeloperoxidase (MPO) activity was used as a marker for neutrophil accumulation in liver samples. Mice were killed, and livers were dissected and perfused through the left ventricle with 20 mL PBS, snap‐frozen in liquid nitrogen and stored at −80°C. MPO activity was measured as described.^28^ In brief, 50 mg liver tissue was homogenized in 50 mmol/L potassium phosphate buffer containing 0.5% hexadecyltrimethyl ammonium bromide and 5 mmol/L ethylenediaminetetraacetic acid. After centrifugation at 12 000 × *g* for 10 minutes at 4°C, the supernatant was incubated in 50 mmol/L potassium phosphate buffer containing the substrate H_2_O_2_ (0.0005%) and *o*‐dianisidine dihydrochloride (167 μg/mL; Sigma‐Aldrich). The enzymatic activity was determined spectrophotometrically by measuring the change in absorbance at 460 nm over 3 minutes.

### 2.11 TUNEL assay

Apoptotic hepatocytes were detected with the in situ terminal deoxynucleotidyl transferase‐mediated dUTP nick‐end labelling (TUNEL) method by use of a TUNEL assay kit (Roche Co., Germany). Liver sections were treated with a mixture of terminal deoxynucleotidyl transferase, digoxigenin‐labelled dUTP and dATP at 37°C for 1 hour and then incubated with peroxidase‐labelled anti‐digoxigenin Ab solution for 30 minutes. Liver tissue sections were analysed by nuclear staining with 500 ng/mL 4’,6‐diamidino‐2‐phenylindole (DAPI) for 10 minutes at room temperature.

### 2.12 Caspase‐9 activity assay

Excised liver tissue was homogenized in T‐PER extraction buffer (Thermo Scientific). Caspase‐9 activity was measured by use of an ELISA kit (Chemicon).

### 2.13 Measurement of NO release

Liver (20 mg) was dissected and homogenized in PBS and then centrifuged at 20800 × g for 10 minutes at 4°C. The accumulation of nitrate and nitrite, stable metabolites of NO, in liver lysates was determined with Griess reagent as previously described.^29^

### 2.14 Statistical analysis

Statistical analysis involved one‐way ANOVA followed by the Fisher least significant difference multiple‐comparison test, as appropriate. The Mann‐Whitney *U* test was used to compare 2 independent groups. Results are presented as mean ± SEM. *P* < 0.05 was considered statistically significant.

## 3 RESULTS

### 3.1 Deletion of Cav‐1 attenuates LPS/GalN‐induced lethality

**Figure 1:**
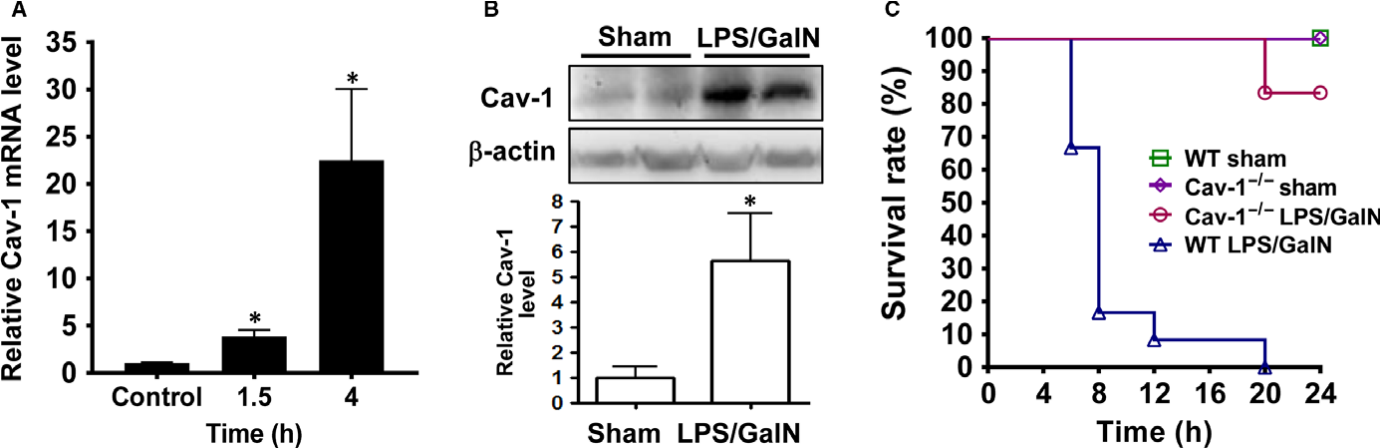
Deletion of Cav‐1 reduces lethality in LPS/GalN‐treated mice. A, Quantitative real‐time PCR of Cav‐1 mRNA level in wild‐type (WT) liver at indicated times after LPS/GalN injection. B, Cav‐1 level in sham‐ and LPS/GalN‐treated liver detected by western blot analysis. Data are mean ± SEM (n = 3). *P < 0.05 vs. control or sham. C, Survival rate (n = 6‐12) of WT and Cav‐1^−/−^ mice intraperitoneally injected with LPS/GalN

Hepatic Cav‐1 was induced after LPS/GalN injection in WT mice. Cav‐1 mRNA level was up‐regulated at 1.5 hours and markedly increased in WT liver at 4 hours after LPS/GalN injection (Figure 1A). As well, Cav‐1 protein level was up‐regulated in WT liver at 6 hours (Figute 1B). After LPS/GalN injection, WT mice began to die within 6 hours, and the death rate reached 100% at 20 hours; in contrast, Cav‐1^−/−^ mice began to die at 20 hours (Figure 1C).

### 3.2 LPS/GalN‐induced liver injury and neutrophil infiltration are attenuated in Cav‐1^−/−^ mice

**Figure 2:**
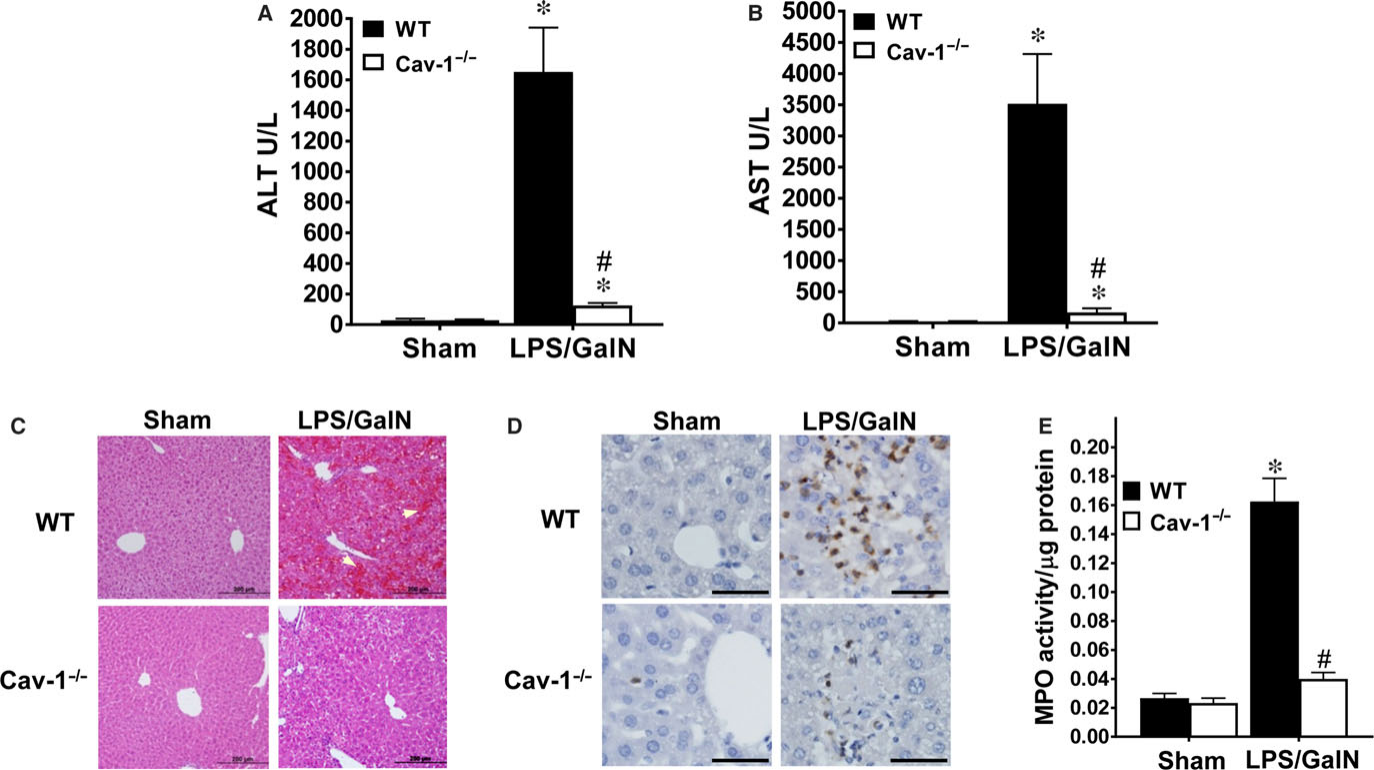
Deletion of Cav‐1 suppresses LPS/GalN‐induced liver damage and neutrophil infiltration. WT or Cav‐1^−/−^ mice were intraperitoneally injected with LPS/GalN for 5 hours. A and B, Plasma levels of alanine aminotransferase (AST) (A) and aspartate aminotransferase (ALT) (B). C‐E, Livers were isolated for histological examination. C, Haematoxylin‐eosin staining of liver sections. Bar, 200 μm. Arrows indicate regions with severe haemorrhage. D, Neutrophils were detected by immunohistochemistry with Gr‐1 antibody in liver. Bar, 50 μm. Images are representative of 6 mice. E, Neutrophil infiltration assessed in liver by myeloperoxidase (MPO) activity assay. Data are mean ± SEM (n = 6). *P < 0.05 vs. sham; ^#^ *P* < 0.05 vs. LPS/GalN‐treated WT mice

Liver damage was examined by serum levels of AST and ALT: both levels were greatly increased at 5 hours after LPS/GalN injection in WT mice (Figure 2A,B). However, these increases were significantly attenuated in Cav‐1^−/−^ mice. Sham‐treated WT and Cav‐1^−/−^ mice showed similar liver histology (Figure 2C). At 5 hours after LPS/GalN administration, WT mice showed severe liver injury, as evidenced by extensive areas of haemorrhage and cell morphology change (Figure 2C). However, Cav‐1^−/−^ mice showed little haemorrhaging and few cells with changed morphology. Next we examined neutrophil infiltration, which is associated with the inflammatory response and damage in acute liver injury.^10^, ^30^ We used Gr‐1 staining and MPO activity to detect neutrophil infiltration. Sham‐treated WT and Cav‐1^−/−^ liver showed no neutrophil infiltration (Figure 2D). LPS/GalN administration increased the number of Gr‐1‐positive cells (Figure 2D) and MPO activity (Figure 2E) in WT liver at 5 hours after treatment, whereas both increases were attenuated in Cav‐1^−/−^ liver.

### 3.3 Deletion of Cav‐1 suppresses LPS/GalN‐induced hepatic cell apoptosis

**Figure 3:**
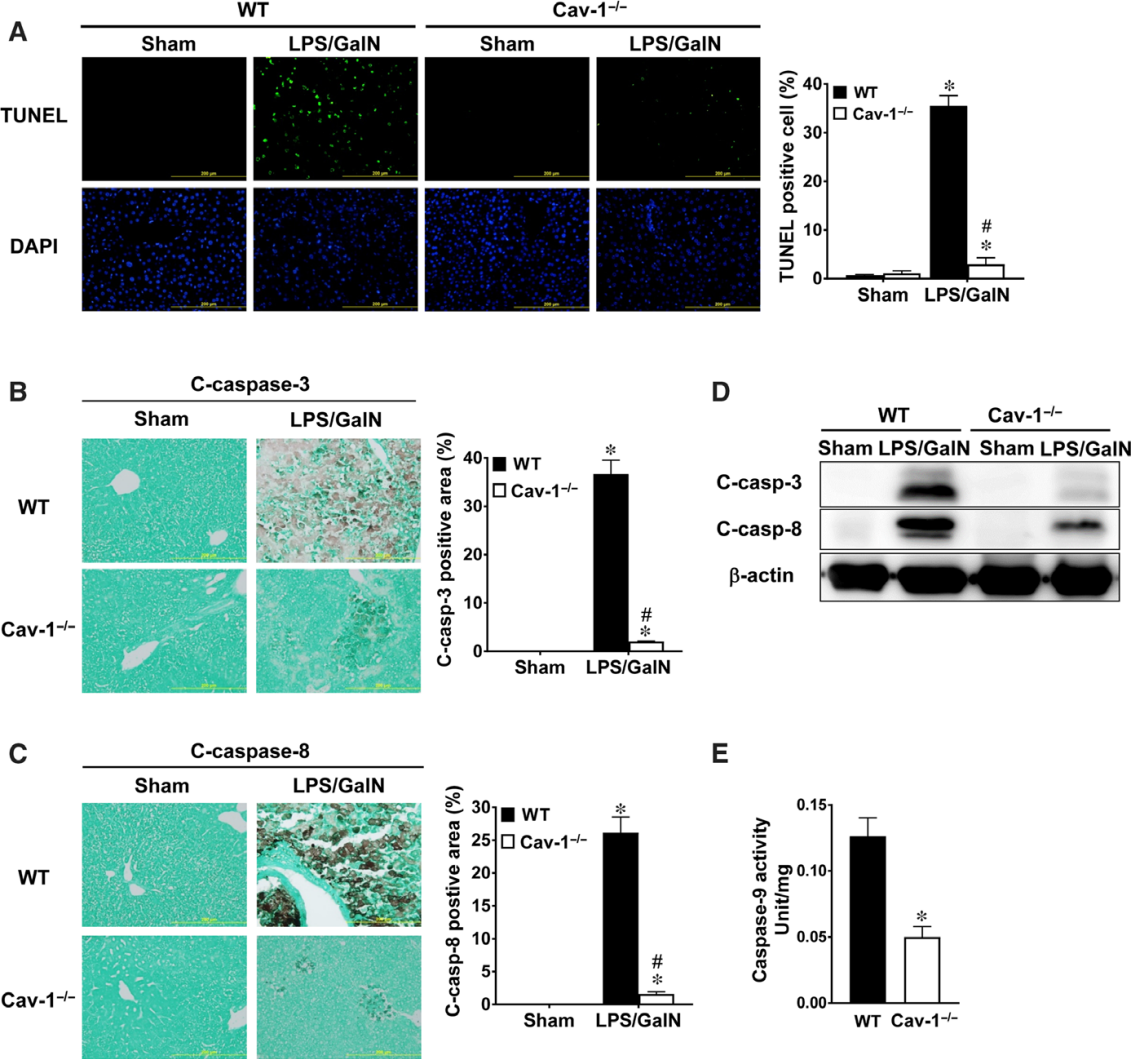
Reduced cell apoptosis and activation of caspase‐3, caspase‐8 and caspase‐9 in livers of LPS/GalN‐treated Cav‐1^−/−^ mice. WT and Cav‐1^−/−^ mice were challenged with LPS/GalN for 5 hours. A, TUNEL staining (green) and confocal microscopy of apoptotic cells in WT and Cav‐1^−/−^ liver. Nuclei were stained with DAPI. B and C,. Immunohistochemistry of cleaved (activated) caspase‐3 (B) and caspase‐8 (C) in livers. Ratio of area of cleaved caspase‐3‐ and caspase‐8‐positive cells were calculated (n = 5). D, Western blot analysis of protein levels of cleaved caspase‐3 and caspase‐8 in liver lysates. E, ELISA of activated caspase‐9 in livers (n = 6). Bar, 200 μm. Data are mean ± SEM. *P < 0.05 vs. WT. ^#^ *P* < 0.05 vs. LPS/GalN‐treated WT mice

Apoptosis of hepatocytes accounts for the major damage to liver in acute liver injury. LPS/GalN‐induced hepatocyte apoptosis was examined by TUNEL staining and caspase activation in WT and Cav‐1^−/−^ liver. WT livers showed a large number of TUNEL‐positive hepatocytes (~35%) at 5 hours after LPS/GalN administration (Figure 3A). However, Cav‐1^−/−^ livers showed fewer TUNEL‐positive hepatocytes (~3%). In addition, sham‐treated WT and Cav‐1^−/−^ livers showed no staining for active caspase‐3 or caspase‐8 (Figure 3B,C). LPS/GalN administration increased the number of both active caspase‐3 and caspase‐8 in hepatocytes of WT mice, whereas this induction was suppressed in Cav‐1^−/−^ mice. Similarly, levels of active caspase‐3 and caspase‐8 were up‐regulated in livers of WT mice treated with LPS/GalN, whereas this induction was suppressed in Cav‐1^−/−^ liver (Figure 3D). Moreover, caspase‐9 activity was lower in livers of Cav‐1^−/−^ mice treated with LPS/GalN than WT mice (Figure 3E). Therefore, deletion of Cav‐1 suppressed LPS/GalN‐induced hepatocyte apoptosis.

### 3.4 Deletion of Cav‐1 suppresses proinflammatory cytokine and chemokine production in LPS/GalN‐treated mice

**Figure 4:**
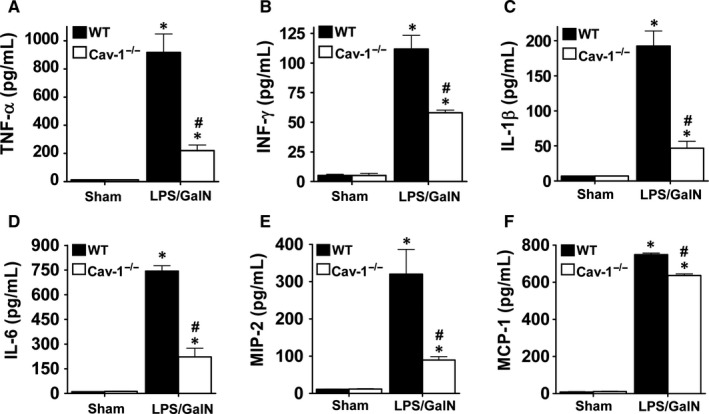
Reduced proinflammatory cytokine and chemokine production in plasma of LPS/GalN‐treated Cav‐1^−/−^ mice. WT or Cav‐1^−/−^ mice were injected with LPS/GalN for 5 hours. Plasma levels of TNF‐α, IFN‐γ, IL‐1β, IL‐6, MIP‐2 and MCP‐1 were measured by ELISA. Data are mean ± SEM (n = 6). *P < 0.05 vs. sham; ^#^ *P* < 0.05 vs. LPS/GalN‐treated WT mice

**Figure 5:**
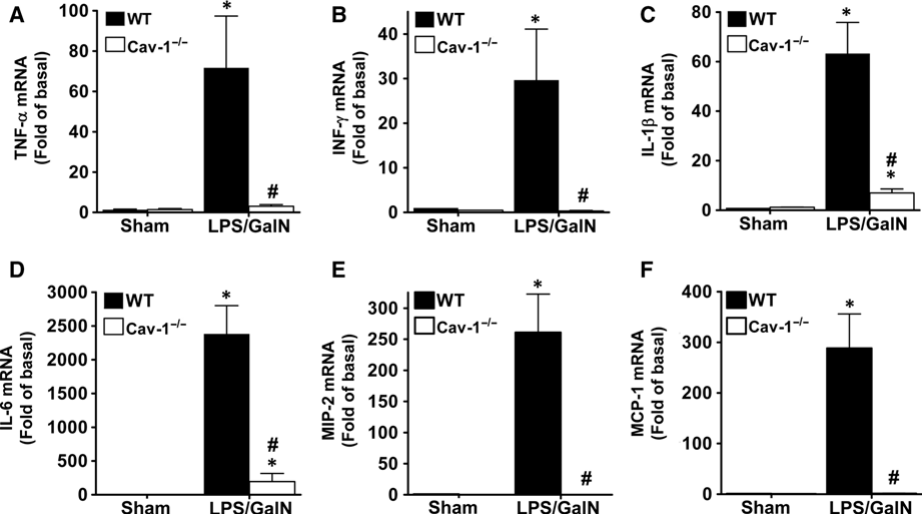
Reduced mRNA levels of proinflammatory cytokines and chemokines in liver of LPS/GalN‐treated Cav‐1^−/−^ mice. WT or Cav‐1^−/−^ mice were injected with LPS/GalN for 5 hours. The mRNA levels of TNF‐α, IFN‐γ, IL‐1β, IL‐6, MIP‐2 and MCP‐1 in livers were measured by real‐time PCR. Data are mean ± SEM (n = 6). *P < 0.05 vs. sham; ^#^ *P* < 0.05 vs. LPS/GalN‐treated WT mice

LPS‐induced proinflammatory cytokine and chemokine expression enhances local inflammation and immune cell infiltration, which is associated with sepsis‐induced liver injury. Plasma levels of TNF‐α, IFN‐γ, IL‐1β, IL‐6, MIP‐2 and MCP‐1 were markedly increased in WT mice at 5 hours after LPS/GalN administration (Figure 4). However, this induction was significantly suppressed in Cav‐1^−/−^ mice. Correspondingly, the mRNA levels of TNF‐α, IFN‐γ, IL‐1β, IL‐6, MIP‐2 and MCP‐1 were markedly induced in liver tissues of WT mice and suppressed in Cav‐1^−/−^ mice (Figure 5). Thus, deletion of Cav‐1 suppressed the LPS/GalN‐induced systemic and hepatic proinflammatory response in mice.

### 3.5 Deletion of Cav‐1 impedes LPS/GalN‐induced TLR4, CD14, VCAM‐1 and ICAM‐1 expression and NF‐κB activation

**Figure 6:**
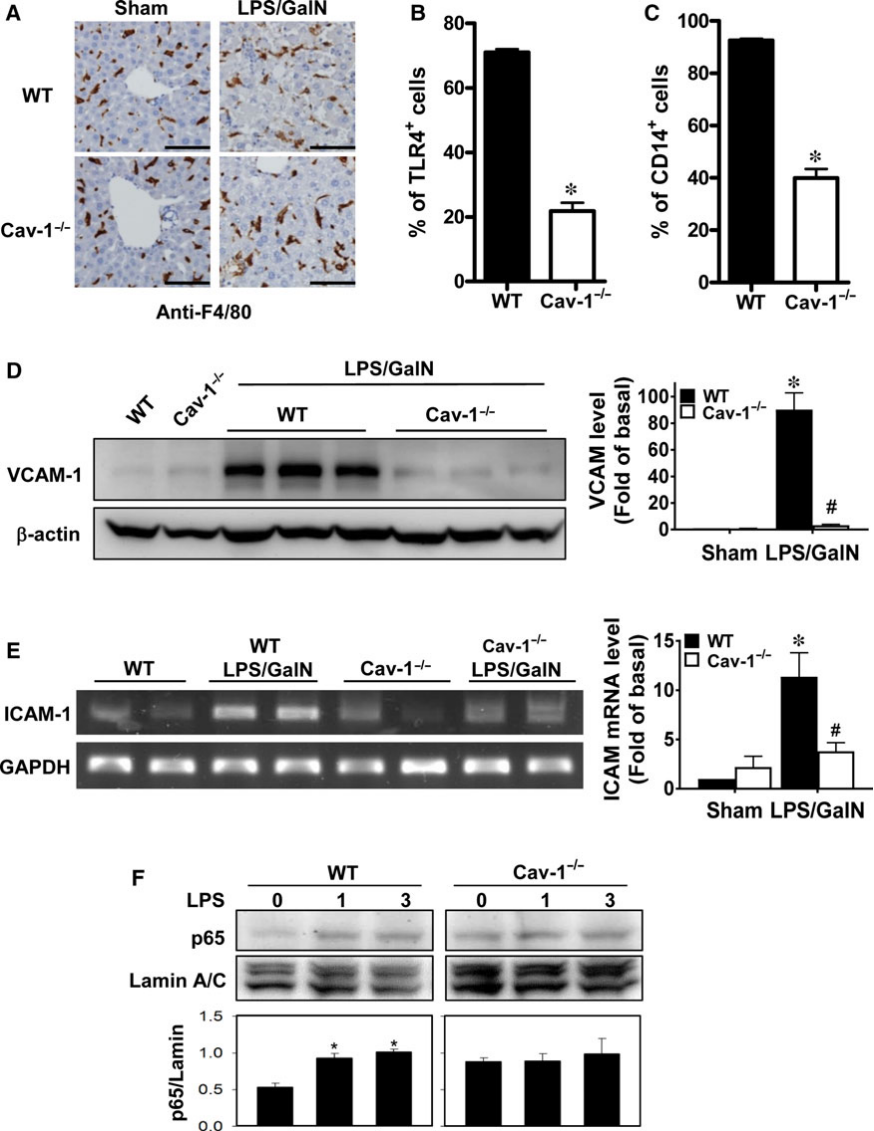
Deletion of Cav‐1 impedes TLR4, CD14, VCAM‐1 and ICAM‐1 expression and NF‐κB activation. A, WT and Cav‐1^−/−^ mouse liver sections were immunostained with anti‐F4/80 antibody to detect Kupffer cells. Bar, 50 μm. Flow cytometry of Kupffer cells isolated from WT and Cav‐1^−/−^ livers and F4/80‐positive cells expressing TLR4 (B) or CD14 (C) with FITC‐labelled anti‐F4/80 antibody plus PE‐labelled anti‐TLR4 or anti‐CD14 antibody. D‐F, WT or Cav‐1^−/−^ mice were injected with LPS/GalN for 5 hours. D, Western blot analysis of protein level of VCAM‐1 in liver. E, RT‐PCR analysis of mRNA level of ICAM‐1 in liver. F, Nuclear fraction of hepatocytes was isolated for western blot analysis of phosphorylated p65 expression. Data are mean ± SEM (n = 5). *P < 0.05 vs. WT or control; ^#^ *P* < 0.05 vs. LPS/GalN‐treated WT mice

We examined whether Cav‐1 is implicated in LPS‐induced CD14‐TLR4 signalling in Kupffer cells. We detected a similar quantity and distribution of Kupffer cells in livers of sham and LPS/GalN‐treated WT and Cav‐1^−/−^ mice (Figure 6A). About 70% and 90% of F4/80‐positive Kupffer cells expressed TLR4 and CD14, respectively, in WT mice (Figure 6B,C). However, Cav‐1^−/−^ mice showed markedly reduced TLR4 and CD14 expression (22% and 40%, respectively), which suggests that Cav‐1 facilitates TLR4 and CD14 expression in Kupffer cells.

Next we examined whether deletion of Cav‐1 altered the expression of adhesion molecules in liver cells, which may help with infiltration of inflammatory cells. VCAM‐1 protein and ICAM‐1 mRNA levels were significantly up‐regulated in WT livers after LPS/GalN treatment, whereas this induction was suppressed in Cav‐1^−/−^ mice (Figure 6D,E).

Because NF‐κB activation is responsible for LPS‐induced inflammatory signalling,^31^ we examined NF‐κB activation by LPS in hepatocytes. Nuclear p65 levels were up‐regulated in WT hepatocytes with LPS induction, but the level of nuclear p65 was not significantly altered in Cav‐1‐deleted hepatocytes (Figure 6F). Thus, deletion of Cav‐1 hindered LPS‐induced NF‐κB activation in hepatocytes.

### 3.6 Deletion of Cav‐1 suppresses LPS/GalN‐induced iNOS expression and NO production in livers

**Figure 7:**
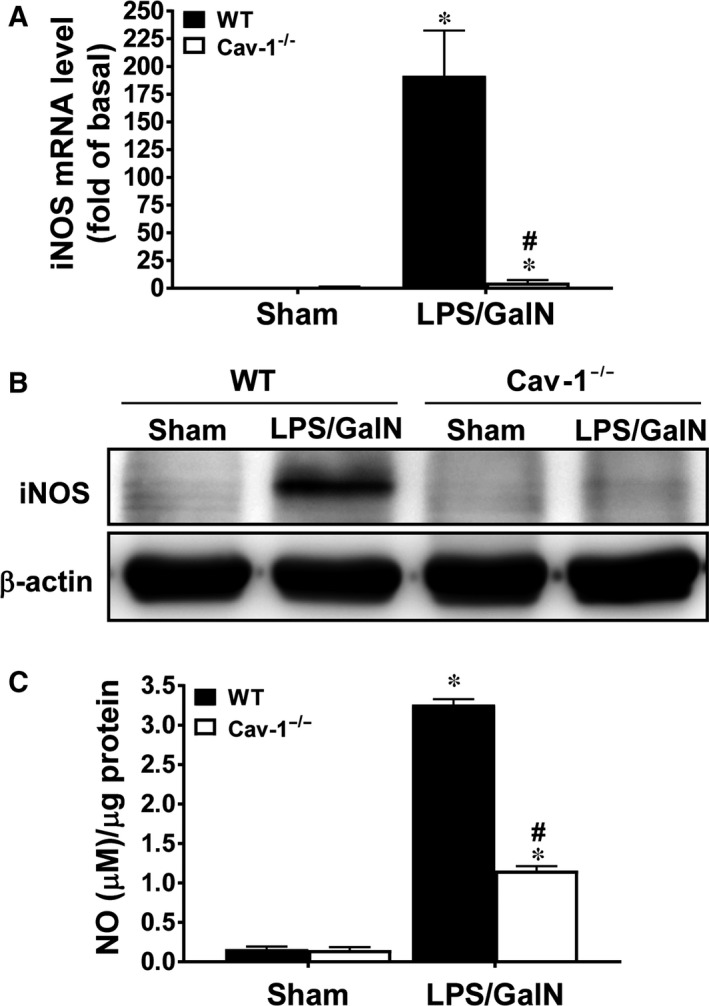
Deletion of Cav‐1 attenuates LPS/GalN‐induced iNOS expression and NO production in mouse livers. WT and Cav‐1^−/−^ mice were injected with LPS/GalN for 5 hours. A, Quantitative real‐time PCR of the iNOS mRNA level in liver. B, Western blot analysis of iNOS protein level in liver. C, Quantification of NO production in liver determined by Greiss reagent. Data are mean ± SEM (n = 6). *P < 0.05 vs. sham group; ^#^ *P* < 0.05 vs. LPS/GalN‐treated WT mice

We examined mouse livers to determine whether deletion of Cav‐1 hindered iNOS induction and NO production, which are implicated in LPS/GalN‐induced hepatic injury.^32^ At 5 hours after LPS/GalN treatment, the mRNA and protein levels of iNOS were markedly up‐regulated in WT livers (~190 and ~22 times, respectively); however, this induction was significantly reduced in Cav‐1^−/−^ mice (~5 and ~1.8 times, respectively) (Figure 7A,B). In parallel, NO production was markedly induced by LPS/GalN in WT livers but was attenuated in Cav‐1^−/−^ livers (~36% of WT) (Figure 7C).

## 4 DISCUSSION

This study demonstrates that deletion of Cav‐1 markedly suppressed the LPS/GalN‐induced inflammatory response, alleviated liver damage and extended mouse survival. Our results suggest that Cav‐1 contributes to the LPS‐induced inflammatory response in that deletion of Cav‐1 suppressed LPS/GalN‐induced hepatic neutrophil infiltration, systematic and hepatic proinflammatory cytokine and chemokine production, NF‐κB activation, and ICAM‐1, VCAM‐1 and iNOS expression in liver. Although deletion of Cav‐1 impairs the monocyte‐to‐macrophage maturation and differentiation,^14^, ^18^ we detected similar Kupffer cell numbers in WT and Cav‐1^−/−^ mouse livers, which suggests that deletion of Cav‐1 does not affect Kupffer cell numbers in liver.

CD14‐TLR4 signalling mediated the LPS‐induced inflammatory response to induce the expression of cytokines, chemokines and adhesion molecules, thereby promoting hepatic immune cell infiltration and an inflammatory response. Inhibition of CD14‐TLR4 signalling by CD14 antisense oligonucleotide or a TLR4 antagonist reduced LPS/GalN‐induced acute liver injury.^8^, ^33^ In macrophages, deletion of Cav‐1 hampered the expression of CD14 and TLR4 and suppressed the Gram‐negative bacteria‐induced inflammatory response.^14^ In line with these results, our data indicate that deletion of Cav‐1 hindered TLR4 and CD14 protein expression in liver Kupffer cells and suppressed the LPS‐induced inflammatory response. Cav‐1 may play a pivotal role in the expression and transportation of TLR4 and CD14 from the endoplasmic reticulum to plasma membrane in maintaining the normal function of Kupffer cells in response to LPS. Because Kupffer cells are major sources of proinflammatory cytokines in the liver's response to LPS,^9^ Cav‐1 knockout may attenuate LPS‐induced CD14‐TLR4 signaling to suppress proinflammatory cytokine and chemokine production and subsequent neutrophil infiltration and liver damage.

Neutrophil infiltration induced by MIP‐2 and MCP‐1 plays an important role in liver inflammation and is also a major cause of liver damage.^34^, ^35^, ^36^ As well, LPS or LPS/GalN induces ICAM‐1 and VCAM‐1 expression in liver cells, which facilitates neutrophil infiltration.^10^, ^11^, ^37^ Deletion of Cav‐1 sequesters neutrophil adhesion to endothelial cells by suppressing NF‐κB activation and ICAM‐1 expression in LPS‐induced lung injury in mice.^16^ Accordingly, suppressed neutrophil infiltration in Cav‐1^−/−^ mouse livers was associated with suppressed MIP‐2, MCP‐1, ICAM‐1 and VCAM‐1 expression. Moreover, LPS/GalN‐induced TNF‐α stimulated the production of pro‐inflammatory cytokines such as INF‐γ and IL‐6 and activated a caspase‐8‐dependent apoptotic signal by binding to TNF receptor.^9^ Overproduction of TNF‐α and other pro‐inflammatory mediators by Kupffer cells can trigger liver inflammation, with a detrimental role in LPS/GalN‐induced liver injury.^9^, ^24^, ^38^ We found that deletion of Cav‐1 suppressed LPS/GalN‐induced TNF‐α, IFN‐γ and IL‐6 expression, which was associated with suppressed hepatic cell apoptosis.

The function of NO in the liver during sepsis is controversial. NO has a protective role after partial hepatectomy^39^ or TNF‐α/GalN treatment.^23^, ^40^ However, high NO production generated by iNOS is a major source of reactive nitrogen species, which can damage a wide range of cellular components in liver during septic shock.^41^, ^42^ Inhibition of iNOS activity by Cav‐1 protected against binge drinking‐induced liver damage by suppressing reactive nitrogen species production.^20^ Deletion of iNOS protected mice against LPS/GalN‐ or Con A‐induced liver damage.^24^, ^32^ Different from ethanol‐induced iNOS expression via the epidermal growth factor receptor/signal transducer and activator of transcription 3 signalling pathway,^20^ our data show that deletion of Cav‐1 hindered LPS‐induced NF‐κB signalling and markedly suppressed iNOS expression and NO production, thus alleviating liver damage. Although the mRNA level of iNOS was barely induced by LPS/GalN in Cav‐1^−/−^ liver, to about 5% of the WT level, a lower but significant amount of NO was generated in Cav‐1^−/−^ liver (36% of the WT level), which is similar to deletion of Cav‐1 suppressing LPS‐induced iNOS and NO production in lung injury in mice.^16^ The unequal induction in mRNA and protein levels for NO production suggests up‐regulated iNOS activity in Cav‐1^−/−^ liver, which is supported by Cav‐1 possibly binding and suppressing iNOS activity.^43^

In conclusion, our results reveal that Cav‐1 plays a critical role in facilitating the inflammatory response mediated by the LPS‐CD14‐TLR4‐NF‐κb pathway in Kupffer cells and hepatocytes. Deletion of Cav‐1 markedly suppressed LPS/GalN‐induced liver inflammation, which led to suppressed liver damage and increased mouse survival. This protective effect was associated with suppressed TLR4 and CD14 expression, NF‐kB activation, VCAM‐1 and ICAM‐1 induction, cytokine and chemokine expression, iNOS expression and NO production. Inhibition of Cav‐1 may provide a novel therapeutic approach for treating LPS‐induced acute liver injury.

## CONFLICT OF INTEREST

The authors confirm that there are no conflicts of interest.
