# Supplementary material for: Deletion of caveolin‐1 attenuates LPS/GalN‐induced acute liver injury in mice
Source: J Cell Mol Med. 2018 Aug 22;22(11):5573–82. doi: 10.1111/jcmm.13831 (PMC6201225; doi:10.1111/jcmm.13831)
Supplement: Supplementary file 1 [file JCMM-22-5573-s001.docx]

Supporting Information Table S1. Primer sequences for RT-PCR and Q-PCR used in this study.

| Gene name | Primer sequence |
| --- | --- |
| Caveolin-1 | GGGCAACATCTAGAAGCCCAACAA  CTGATGCACTGAATCTCAATCAGGAA |
| TNF-α | CAAATGGCCTC CCTCTCATC AG  GTGGGTGAGGAGCACGTAGTC |
| INF-γ | TACTGCCACGGCACATGCATTGAA  TGCAGGATTTTCATGTCACCAT |
| IL-1β | TACAAGGAGAGACAAGCAACGACA  GATCCACACTCTCCAGCTGCA |
| IL-6 | ACCACTCCCAACAGACCTGTC  ACTCCAGGTAGCTATGGTACTC |
| MIP-2 | GCGCCCAGACAGAAGTCATAG  AGCCTTGCCTTTCTTCAGTTACGG GT |
| MCP-1 | TTAAAAACCTGGATCGGAACCAA  GCA TTAGCTTCAGATTTACGGGT |
| iNOS | CAGCTGGGCTGTACAAACCTT  CATTGGAAGTGAAGCGTTTCG |
| β-actin | CGACAACGGCTCCGGCATGTG  GGTCTCAAACAT GATCTGGG |
| ICAM | TTCACACTGAATGCCAGCTC  GTCTGCTGAGACCCCTCT TG |
| GADPH | ATGTGTCCGTCGTGGATCTG  CCTCAGTGTAGCCCAAGATG-3’ |
